# Targeting T-cell integrins in autoimmune and inflammatory diseases

**DOI:** 10.1093/cei/uxad093

**Published:** 2023-08-09

**Authors:** Aidan J Kelly, Aideen Long

**Affiliations:** Trinity Translational Medicine Institute, Trinity College Dublin, Trinity Centre for Health Sciences, St James’s Hospital, Dublin D08 NHY1, Ireland; Trinity Translational Medicine Institute, Trinity College Dublin, Trinity Centre for Health Sciences, St James’s Hospital, Dublin D08 NHY1, Ireland

**Keywords:** T cells, integrins, autoimmune, inflammatory bowel disease, multiple sclerosis, psoriasis

## Abstract

The recruitment of T cells to tissues and their retention there are essential processes in the pathogenesis of many autoimmune and inflammatory diseases. The mechanisms regulating these processes have become better understood over the past three decades and are now recognized to involve temporally and spatially specific interactions between cell-adhesion molecules. These include integrins, which are heterodimeric molecules that mediate in-to-out and out-to-in signalling in T cells, other leukocytes, and most other cells of the body. Integrin signalling contributes to T-cell circulation through peripheral lymph nodes, immunological synapse stability and function, extravasation at the sites of inflammation, and T-cell retention at these sites. Greater understanding of the contribution of integrin signalling to the role of T cells in autoimmune and inflammatory diseases has focused much attention on the development of therapeutics that target T-cell integrins. This literature review describes the structure, activation, and function of integrins with respect to T cells, then discusses the use of integrin-targeting therapeutics in inflammatory bowel disease, multiple sclerosis, and psoriasis. Efficacy and safety data from clinical trials and post-marketing surveillance are presented for currently approved therapeutics, therapeutics that have been withdrawn from the market, and novel therapeutics currently in clinical trials. This literature review will inform the reader of the current means of targeting T-cell integrins in autoimmune and inflammatory diseases, as well as recent developments in the field.

## Teaser

Activated T cells bind to the endothelium using activated integrins as part of the multi-step process of extravasation. The expression of specific repertoires of adhesion molecules on T cells and the endothelium mediates spatial and temporal specificity of extravasation. The role of integrins in the extravasation of activated T cells can be therapeutically inhibited in multiple sclerosis, psoriasis, and inflammatory bowel disease.

## Graphical Abstract

**Graphical Abstract:**
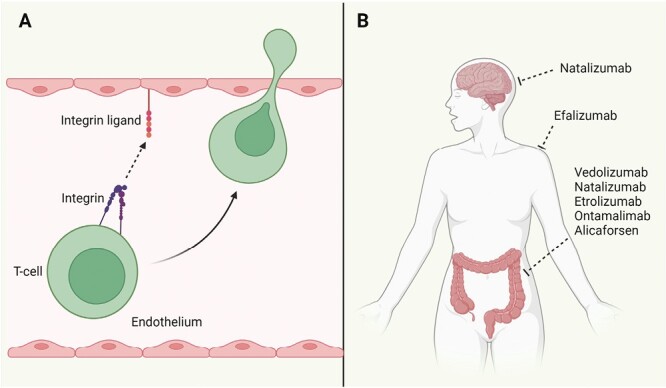

## Introduction

Integrins are a class of cell-adhesion molecules that are expressed on the cell surface membrane and bind components of the extracellular matrix and other integrin ligands. By further associating with intracellular molecules, integrins mediate signaling from the extracellular environment to the intracellular environment—out-to-in signalling—and from the intracellular environment to the extracellular environment—in-to-out signaling [1]. Integrins expressed by circulating leukocytes, including T cells, mediate the process of extravasation which allows these leukocytes to gain access to tissues where they act [2], and differential repertoires of integrins expressed by T cells confer specificity with regard to the site of extravasation [3]. In the last three decades, therapeutic agents that target T-cell integrins have been introduced for the treatment of autoimmune and inflammatory diseases, including inflammatory bowel disease (IBD), multiple sclerosis (MS), and psoriasis [4]. This literature review will briefly describe the structure, activation, and function of integrins with regard to T-cell activity, then will discuss the use of therapeutic agents that target T-cell integrins in autoimmune and inflammatory diseases.

## Integrin structure and activation

Integrins are heterodimers composed of two non-covalently associated glycoprotein subunits, denoted α-chain and β-chain, and can exist in inactive, primed, or active states. There are 18 isoforms of the α-chain and 8 isoforms of the β-chain, and 24 combinations of α- and β-chains exist [1]. The priming of integrins for ligand-binding is mediated by the binding of cytoplasmic adaptor proteins, such as talin and kindlin, to the cytoplasmic tail of the β-chain, causing the bent extracellular portion of the heterodimer to straighten [5]. Ligand-binding completes integrin activation. Campbell and Humphries [6] provide a more detailed description of the structure and activation of integrins.

## Leukocyte extravasation

Leukocyte extravasation is the process by which leukocytes leave the circulation to enter the surrounding tissue, and depends on the expression of adhesion molecules [7]. To explain the spatial and temporal specificity and diversity of extravasation, the multistep paradigm was proposed by Butcher [2], consisting of leukocyte rolling, activation, and arrest. The combinatorial use of molecular components of the multistep paradigm of extravasation can be conceptualized as an “area code” that determines the migratory characteristics of particular leukocytes [3].

The initial interaction of leukocytes and endothelial cells is mediated by transient interactions between selectins and their ligands, which are upregulated in response to mediators of acute inflammation, including interleukin-1 (IL-1) and tumour necrosis factor alpha (TNF-α) [3]. Selectin-mediated tethering causes rolling of leukocytes along the endothelium to slow them down and allow further interactions to occur. A further effect of selectin binding is to cause the initial in-to-out activation of leukocyte integrins via downstream signalling pathways involving Src family kinases, phospholipase C-γ2, and Rap1, a small GTPase [8]. Integrins can also contribute to leukocyte rolling [9].

Chemokines are small polypeptides that are secreted by a wide variety of cell types as part of the acute inflammatory response [10]. Endothelial cells are induced to express chemokines on their luminal surface in response to inflammatory cytokines and activated platelets generate chemokines by proteolysis for subsequent deposition on the endothelial surface, including CCL5, CXCL4, and CXCL5 [9]. Chemokines act via heterotrimeric G-protein-coupled receptors (GPCRs) to initiate downstream signalling cascades; one result of this is the binding of intracellular adaptor proteins, e.g. talin-1, kindlin-3, cytohesin-1, and 14-3-3 family members, with the cytoplasmic tails of integrin β-chains, possibly mediated by RhoA and Rac1 GTPases, resulting in integrin activation [8].

Once leukocyte integrins are activated, they bind to their cognate integrin ligands present on the endothelium. αLβ2 binds to ICAM-1, which is constitutively expressed at low levels and upregulated as part of the inflammatory response [11]. This binding causes firm adhesion of the leukocyte to the endothelium due to the assembly of signalosomes which recruit protein tyrosine kinases involved in further downstream signalling [9]. Other integrins which are expressed exclusively by leukocytes also bind to their ligands to promote this process of firm adhesion.

Following firm adhesion, leukocytes crawl along the endothelial surface to identify a point for transmigration across the endothelium. This occurs by an integrin-dependent process that mediates cytoskeletal changes in both the leukocyte and endothelial cells. For example, α4β1 binds to VCAM-1 on the endothelial cell, causing paxillin to bind to the cytoplasmic tail of the α4-chain [12]. Paxillin acts to localize the activity of Rac GTPase to the leading edge of the cell, causing the formation of a lamellipodium and promoting leukocyte crawling [13]. Transmigration is completed by migration through the endothelial basement membrane, facilitated by proteases such as matrix metalloproteinases and neutrophil elastase [9].

## Lymphocyte homing

Naïve T cells continuously circulate through the lymph nodes (LNs). Entry into LNs is primarily via high endothelial venules (HEVs) and follows the multistep paradigm of extravasation. Tethering and rolling of naïve T cells occurs via the binding of l-selectin to peripheral node addressin; activation of integrins in naïve T cells occurs via the binding of chemokines CCL21 and CCL19 to CCR7; and firm adhesion occurs via the binding of αLβ2 to ICAM-1 [14, 15]. The binding of naïve T cells to HEVs in mesenteric LNs and Peyer’s Patches follows a similar mechanism but is more dependent on α4β7-MAdCAM-1 binding for rolling and firm adhesion [14].

When naïve T cells are induced to differentiate into effector T cells following antigen presentation by dendritic cells (DCs), changes to their repertoire of cell-adhesion molecules, including integrins, allow the development of lymphocyte homing, that is, the tendency for lymphocytes to home to the site whence their stimulating antigen was derived [16]. For example, CD103^+^ DCs in the mesenteric LNs (MLNs), which derive from CD103^+^ DCs in the small intestine lamina propria, have been shown to induce FoxP3^+^ T-cell differentiation and gut-homing receptor expression, including CCR9 and α4β7, via retinoic acid signalling [17]. In the lamina propria, transforming growth factor-β1 (TGF-β1) signalling further contributes to the development of gut-homing T cells by increasing the expression of αE- and β7-integrin chains [18]. This example of the development of gut-homing T cells is illustrative of the role of DCs and the LN microenvironment in the development of T-cell tropism.

## Inflammatory bowel disease

### Pathophysiology

IBD is a chronic inflammatory disease of the gastrointestinal tract encompassing ulcerative colitis (UC) and Crohn’s disease (CD). UC is relapsing, non-transmural, and restricted to the colon; CD is relapsing, transmural, and can affect the entire gastrointestinal tract, from mouth to anus [19]. The clinical features of UC and CD differ but commonly include abdominal pain, diarrhoea, bloody stools, and weight loss [20]. The pathogenesis of UC and CD is complex and still unknown but involves genetic factors, gut microbial factors, other environmental factors, and dysregulation of the innate and adaptive immune systems [20]. Risk loci correlated with expression changes in integrins or integrin ligands have been identified by genome-wide association studies (GWAS) [21]. The interplay of these factors is posited to allow the translocation of antigens derived from the contents of the intestinal lumen across the dysfunctional epithelial barrier to cause local activation of the immune system with subsequent structural changes to the intestinal wall [22].

CD4 + Th cells are known to regulate the type of adaptive immune response that takes place via cytokine signalling. Crohn’s disease is skewed towards a Th1- and Th17-cell response, while ulcerative colitis is skewed towards a Th2-, Th17-, and Th9-cell response [20]. Inducible Treg (iTreg)-cell numbers are increased in inflamed intestinal lamina propria, where they retain their suppressive function [23]. However, it has been shown that effector T cells exhibit resistance to this suppression in IBD, suggesting that it is this that blunts the effectiveness of iTreg cells [24]. Perturbations in T-cell plasticity may also contribute to the development of IBD by upsetting the intestinal immune homeostasis [25].

MAdCAM-1 is expressed in the HEVs of the small intestine, Peyer’s patches, and large intestine, and interacts with l-selectin and α4β7 to mediate rolling and firm adhesion of T cells, respectively [14]. Its expression is increased at sites of active inflammation in IBD [26]. VCAM-1, which is expressed only when endothelial cells are stimulated by pro-inflammatory cytokines, also binds α4β7 (with lower affinity than MAdCAM-1 does) as well as α4β1. As MAdCAM-1 expression is lower in the large intestine than in the small intestine, it is likely that VCAM-1 plays a greater role in lymphocyte homing to the former [27]. ICAM-1 is also expressed by intestinal endothelial cells and interacts with αLβ2 and αMβ2. CCR9-CCL25 binding promotes homing to the small intestine, while CCR10-CCL28 binding promotes homing to the large intestine [28, 29]. E-cadherin is expressed by intestinal epithelial cells on their basolateral surface and interacts with αEβ7 to cause the retention of tissue-resident memory T cells in the lamina propria; these cells have been shown to orchestrate the recruitment of other pro-inflammatory leukocytes, including myeloid cells [30].

**Figure 1.**
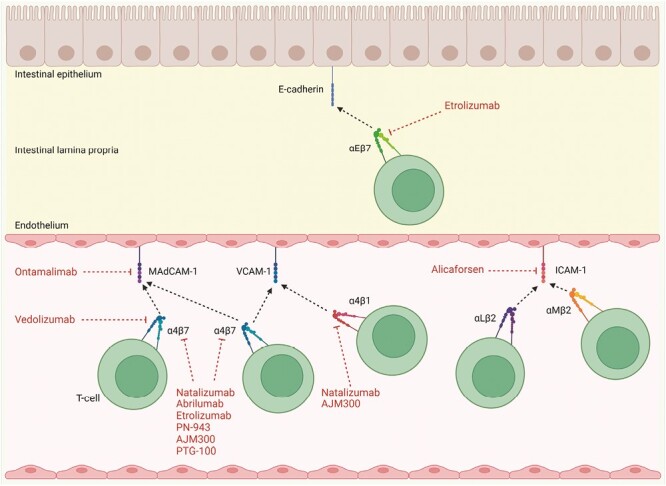
Targeting T-cell integrins in inflammatory bowel disease. Vedolizumab and ontamalimab inhibit the binding of α4β7 to MAdCAM-1 by inhibiting each molecule, respectively. Natalizumab, abrilumab, etrolizumab, PN-943, AJM300, and PTG-100 inhibit the binding of α4β7 to MAdCAM-1 and VCAM-1 by inhibiting the integrin itself. Natalizumab and AJM300 also inhibit the binding of α4β1 and VCAM-1. Alicaforsen downregulates the expression of ICAM-1 to inhibit its binding to αLβ2 and αMβ2. The action of the aforementioned drugs is to inhibit extravasation of T cells in the gut. Etrolizumab also acts to reduce the retention of T cells in the intestinal lamina propria by inhibiting the binding of αEβ7 to E-cadherin, expressed by intestinal epithelial cells. Created with BioRender.com

The ‘area code’ used to direct T-cell homing to the gut presents several therapeutic targets for IBD. Thus, drugs that inhibit the action of α4β7, α4β1, and αEβ7 have been developed, trialled, and authorized for the treatment of IBD (Fig. 1) [27]. Comparative efficacy and safety data for these drugs are summarized in Table 1.

**Table 1.** Comparative efficacy and safety data of drugs targeting T-cell integrins for which phase-III trials have been carried out for UC, CD, MS, and/or psoriasis

Drug	Target	Disease	Efficacy	Comparator	Confirmed PML risk
Vedolizumab	α4β7	Moderately to severely active UC	Induction and maintenance of clinical remission [31]	Placebo	No
		Moderately to severely active CD	Induction and maintenance of clinical remission [32]		
Natalizumab	α4β1, α4β7	Moderately to severely active CD	Induction of clinical response [33]	Placebo	Yes [34]
		Active CD	Maintenance of clinical response [34]		
		RRMS	Reduction of annual relapse rate and risk of sustained progression of disability [35]		
		SPMS	Reduction of upper limb progression of disability [36]		
Natalizumab + interferon β-1a	α4β1, α4β7	RRMS	Reduction of annual relapse rate and risk of sustained progression of disability [37]	Interferon β-1a	Yes [34]
Etrolizumab	αEβ7, α4β7	Moderately to severely active UC	Induction of clinical remission [38, 39]	Placebo	No
		Moderately to severely active CD	Maintenance of clinical remission following failed TNF-α inhibitor therapy [40]		
Alicaforsen	ICAM-1	Chronic pouchitis	None [41]	Placebo	No
		Active CD	None [42]		
AJM300	α4β1, α4β7	Moderately active UC	Induction of clinical response following failed mesalazine therapy [43]	Placebo	No
Efalizumab	αLβ2	Moderate to severe psoriasis	Induction and maintenance of clinical response [44]	Placebo	Yes [45]

### Vedolizumab

Vedolizumab is an anti-α4β7 IgG1 monoclonal antibody that is the humanized version of the murine Act-1 monoclonal antibody [46]. Act-1 showed anti-inflammatory effects and improved stool consistency when administered to chronically colitic cotton-top tamarins [47]. Vedolizumab binds to an epitope within the β7-chain of the α4β7 heterodimer and therefore would be expected to also bind to, and inhibit, αEβ7 [46]. However, this is not the case, likely due to the presence of an α-I domain in αEβ7 [48]. Vedolizumab binding to α4β7 inhibits the interaction of α4β7 with MAdCAM-1 and fibronectin, but not VCAM-1, as VCAM-1 binds to α4β7 without clashing with vedolizumab [48]. Vedolizumab binding to α4β7 induces the internalization of α4β7, and removal of vedolizumab restores α4β7 expression and MAdCAM-1 binding to normal within 4 days, *in vitro*. As the elimination half-life of vedolizumab is 25.5 days, the restoration of α4β7 expression and MAdCAM-1 binding *in vivo* should not occur between doses (usually 4- to 8-weekly) [46]. As described above, α4β7 is specific to gut-homing lymphocytes. Preclinical and phase-I trials of vedolizumab demonstrated that its mechanism of action is gut-specific by showing a reduction in α4β7 T cells in the lamina propria, an increase in α4β7 T cells in the peripheral vasculature, an attenuated immune response to enteral antigen challenge, and a conserved immune response to parenteral antigen challenge [49, 50]. However, alternative hypotheses for the mechanism of action of vedolizumab have been proposed; a longitudinal, observational study by Zeissig *et al*. [51] in patients with IBD showed that vedolizumab had little impact on the abundance and phenotype of lamina propria T cells, but caused substantial changes to the innate immune system, including in the expression of pattern recognition receptors, chemokines, and innate effector molecules, and in the abundance and phenotype of lamina propria macrophages.

A phase-III, randomized controlled trial (RCT) for vedolizumab in patients with active, moderate to severe UC demonstrated efficacy for induction of clinical remission and for maintenance therapy; the frequency of adverse events was similar in the vedolizumab and placebo groups [31]. A phase-III RCT in patients with active, moderate to severe CD demonstrated efficacy for induction of clinical remission and for maintenance therapy; the incidence of serious adverse events was higher in the vedolizumab group than in the placebo group, including infections and serious infections [32]. A Phase-III, RCT in patients with active, moderate to severe CD in whom previous therapy with TNF-α inhibitor had failed did not meet its primary endpoint of induction of clinical remission at week 6, but demonstrated efficacy for induction of clinical remission at week 10; the frequency of adverse events was similar in the vedolizumab and placebo groups [52]. Post hoc analysis of these trials found a positive exposure–efficacy relationship between vedolizumab average concentration and clinical remission rates, suggesting that dosing of vedolizumab should be considered as a reason for poor response and adjusted, if necessary [53]. Indeed, if clinical response is lost during vedolizumab maintenance therapy, dose intensification can restore it in more than half of cases [54]. Multiomics analysis has recently shown that low pre-treatment serum vitamin D concentration can predict the future failure of vedolizumab, a result that should inspire further research into this field, including the effect of vitamin D supplementation on vedolizumab failure [55].

A review of the literature on the use of vedolizumab in IBD suggested that it is effective for induction and maintenance of remission in patients with moderately to severely active UC or CD who have had an inadequate response with, lost response to, or were intolerant to either conventional therapy or a TNF-α inhibitor, and is a first-line alternative to TNF-α inhibitor therapy in UC [56]. This is in line with guidance from the National Institute for Health and Care Excellence (NICE) [57, 58].

There have been conflicting reports on the effect of vedolizumab on the extra-intestinal manifestations of IBD, particularly spondyloarthritis, which is present in up to 30% of patients with IBD [59]. One case series described new-onset and exacerbation of spondyloarthritis following vedolizumab treatment for IBD, possibly due to “drifting” of circulating α4β7 + T cells [59]. A later prospective, observational study of 53 patients who began vedolizumab treatment for IBD reported no new-onset or exacerbation of spondyloarthritis but showed improvements in existing spondyloarthritis in some patients [60]. Post hoc analyses of phase-III trials of vedolizumab suggest that vedolizumab reduces the risk of new and worsening arthralgia in CD compared to placebo, with no effect in UC [61].

### Natalizumab

Natalizumab is a humanized anti-α4 IgG4 monoclonal antibody which binds to the β-propeller and thigh domain of the α4-chain of α4β1 and α4β7 [62]. As occurs in vedolizumab, the binding of α4β7 by natalizumab inhibits its binding to its ligands, MAdCAM-1 and VCAM-1, and thereby inhibits the homing of T cells to the lamina propria, MLNs, and Peyer’s patches of the intestines. The binding of α4β1 by natalizumab inhibits its binding to VCAM-1, potentiating the inhibition of T-cell gut-homing. The effects of natalizumab on integrin binding to ligands have been demonstrated *in vitro* [63].

A phase-III trial (ENACT) of natalizumab in patients with active CD demonstrated that natalizumab was not efficacious in achieving clinical response or remission at week 10, compared to placebo, but that it was efficacious in maintaining clinical response through week 36, compared to placebo [34]. The rate of serious adverse events was similar in each group; however, in an open-label extension study, one patient treated with natalizumab died from progressive multifocal leukoencephalopathy (PML) [see Box 1; [34]. Another phase-III RCT (ENCORE) of natalizumab in patients with moderately to severely active CD demonstrated efficacy in achieving clinical response and remission at week 8 sustained through week 12 [33]. A pilot study of natalizumab in 10 patients with active UC demonstrated efficacy and tolerability [63]. Phase-III RCTs for natalizumab in UC have not taken place.

Box 1: Progressive multifocal leukoencephalopathy

Progressive multifocal leukoencephalopathy (PML) is a severe and frequently fatal brain infection caused by the John Cunningham virus (JCV), a human polyomavirus that causes lysis of infected glial cells leading to demyelination [64]. An analysis of data from post-marketing sources, clinical studies, and an independent Swedish registry found that the risk of PML associated with natalizumab treatment is increased by three factors: anti-JCV antibody positive titres; prior use of immunosuppressants; and duration of natalizumab therapy [65]. These risk factors should be considered when prescribing these drugs [66].

Due to the risk of PML, natalizumab was withdrawn from the market in 2005. In the USA, natalizumab was reintroduced for moderately to severely active CD in patients who have failed conventional and TNF-α inhibitor therapy; it is only available through a risk evaluation and mitigation strategy called the TOUCH Prescribing Program [67]. It is not authorized for the treatment of CD in Europe, being only rarely used in patients with severe concomitant multiple sclerosis [68].

### Etrolizumab

Etrolizumab is a humanised anti-β7 monoclonal antibody and, as such, binds to α4β7 and αEβ7 to inhibit their binding to MAdCAM-1 and VCAM-1, and E-cadherin, respectively. T cells expressing αEβ7 bind to E-cadherin that is expressed by epithelial cells on their basolateral surface, causing retention of these T cells in the lamina propria [30]. αEβ7 is highly expressed by CD8^+^ and Th9 cells in patients with IBD, and this expression is induced by TGF-β signalling; in contrast, α4β7 is more highly expressed by Th2- and Th17 cells [69]. As expected from this observation, treatment with etrolizumab reduced colonic CD8^+^ and Th9 cells in a dextran sodium sulphate (DSS)-induced colitis immunodeficient mouse models by a greater degree than vedolizumab, likely by reducing retention of these cells in the lamina propria [69]. αEβ7 tissue-resident memory CD8^+^ T cells are also present in the lungs and the brain [70, 71], so it could be reasonably expected that etrolizumab would reduce their presence in these tissues. However, animal studies have shown that etrolizumab has no effect on lymphocyte homing to the brain [72]. Another mechanism of action of etrolizumab may be the inhibition of αEβ7 on colonic DCs. These DCs ordinarily generate regulatory T cells but seem to have a more pro-inflammatory function in UC [73].

Etrolizumab underwent extensive testing in phase III RCTs in participants with UC and CD, and in comparison with placebo, infliximab, and adalimumab [38–40, 74, 75]. These largely failed their primary endpoints, except HICKORY, which compared etrolizumab to placebo for inducing clinical remission at week 14 in patients with moderately to severely active UC who had failed TNF-α inhibitor therapy [38]; HIBISCUS I, which compared etrolizumab to placebo for inducing clinical remission at week 10 in patients with moderately to severely active UC who were naïve to TNF-α inhibitor therapy [39]; and BERGAMOT, which compared etrolizumab to placebo for maintaining clinical remission and endoscopic improvement at week 66 in patients with moderately to severely active CD who had failed TNF-α inhibitor therapy and who had clinical response at week 14 [40]. Etrolizumab was well tolerated in all trials. Retrospective analysis of data from phase-II trials of etrolizumab in patients with UC showed that those with a higher colonic expression of granzyme A messenger RNA (mRNA) and integrin αE mRNA had a greater likelihood of achieving clinical remission, showing that these are potential biomarkers for patients who would benefit from etrolizumab [76]. Despite this, due to the disappointing phase III trials, it is unlikely that etrolizumab will achieve market approval.

### Ontamalimab

Ontamalimab is a human anti-MAdCAM-1 IgG2K monoclonal antibody; it is also known as SHP647 and PF-00547659. MAdCAM-1 is the cognate ligand of α4β7 and therefore acts to mediate the rolling and firm adhesion steps of extravasation of α4β7-expressing leukocytes [14]. It is also a ligand for l-selectin, mediating rolling of lymphocytes in the gut-associated lymphoid tissue. MAdCAM-1 expression is increased at sites of intestinal inflammation, and MAdCAM-1 is also expressed at extra-intestinal sites that can be affected in patients with IBD, such as the joints, eyes, and skin [77].

A phase-II RCT of ontamalimab in patients with moderately to severely active UC who had failed conventional therapy demonstrated efficacy in inducing clinical remission, with the greatest effect being at 22.5 mg and 75 mg doses in patients who had not received previous TNF-α inhibitor therapy [78]. A phase-II RCT of ontamalimab in patients with moderately to severely active CD who had failed TNF-α inhibitor therapy and/or conventional therapy did not demonstrate efficacy in causing a clinical response despite an increase in circulating β7^+^ CD4^+^ T cells [79]. The clinical trial development program was terminated by Takeda Pharmaceutical Company Limited following its acquisition of Shire plc [80].

### Alicaforsen

Alicaforsen is an antisense oligonucleotide against ICAM-1 mRNA which acts to reduce its expression; ICAM-1 expression is increased in inflammation [11]. Alicaforsen enema has shown efficacy in patients with mildly to moderately active distal UC, compared to placebo [81]. It showed no efficacy compared to placebo in a phase-III trial for chronic pouchitis [41]. Parenteral alicaforsen has not shown efficacy in CD [42].

### Future therapies

Future therapies that target T-cell integrins in IBD are in development. Abrilumab is an anti-α4β7 IgG2 monoclonal antibody that has demonstrated efficacy compared to placebo in phase-II trials for UC but not CD; phase-III trials have not been registered [67]. PN-943 is an orally administered, gastrointestinal-restricted peptide that binds to α4β7 [82]. Phase-II trials in patients with UC are ongoing. AJM300 is an orally administered α4 antagonist, thereby inhibiting the binding of α4β1 and α4β7 to their ligands (the same mechanism of action as natalizumab). A phase-I trial in healthy males showed that it increased the circulating lymphocyte count [83]. A phase-III trial in patients with moderately active UC who had failed mesalazine demonstrated efficacy in achieving clinical response compared to placebo; the incidence of adverse events was similar in each group [43]. PTG-100 is an oral α4β7 antagonist peptide that has shown promising phase-IIa efficacy and safety data [84].

## Multiple sclerosis

### Pathophysiology

MS is a chronic, autoimmune disease of the CNS. A total of 85% of patients begin with relapsing-remitting MS (RRMS), characterized by clinical relapses followed by functional recovery to near the baseline. In total 50% of RRMS patients develop progressive disease, termed secondary progressive MS (SPMS). Most of the other 15% of MS patients have progressive disease from the start, termed primary progressive MS (PPMS) [85]. The clinical presentation of MS is heterogeneous and can include sensory and visual disturbances, motor impairments, fatigue, pain, and cognitive deficits [86]. The pathogenesis of MS is complex and not completely understood, but involves genetic factors, environmental factors, and dysregulation of the immune system. In populations of northern European origin, the strongest risk alleles are those in the HLA class II region of chromosome 6; *VCAM-1* is also a risk locus [87]. Environmental factors include a history of Epstein-Barr virus infection, tobacco use, and alterations in the gut microbiota [85].

In health, the CNS is an “immune-privileged” site with low entry of lymphocytes [88]. Infiltration by immune cells of the CNS across the blood-brain barrier (BBB) and blood-CSF barrier in the choroid plexus characterizes the early, acute phases of MS. Macrophages are the dominant cells at this stage, followed by CD8^+^ T cells, CD4^+^ T cells, and B cells [86]. Naïve CD4^+^ T cells that infiltrate the CNS are activated by DCs in experimental autoimmune encephalitis (EAE) leading to the activation of monocytes and the initiation of epitope spreading to cause the activation of other naïve CD4^+^ T cells [89]. Th1- and Th17-cells are the dominant pro-inflammatory CD4^+^ T-cell subsets, while Th2-cells may be associated with treatment response [90]. Myelin-specific CD8^+^ T cells are activated by epitope spreading due to cross-presentation by DCs; these T cells are more prevalent in MS lesions than CD4^+^ T cells and are closely associated with neurodegeneration at all stages of disease progression [86, 91]. Activated macrophages and microglia promote demyelination throughout the disease course due to free radical-mediated damage from NADPH oxidase activation [92]. The activity of these innate immune cells is maintained by the production of CCL2 and granulocyte-macrophage colony-stimulating factor by astrocytes [86]. Later in the disease progression, while CNS inflammation continues, infiltration by immune cells across the BBB decreases, suggesting that CNS inflammation becomes self-sustaining, and raising important considerations for the use of therapies that target lymphocyte extravasation, including anti-integrin therapies [91]. In SPMS, meningeal tertiary lymphoid structures, formed of aggregated plasma cells, B cells, T cells, and DCs, may contribute to inflammation [86].

The initial interaction of T cells with BBB endothelial cells is the binding of α4β1 to VCAM-1; this interaction is strengthened by chemokine GPCR-dependent signalling [88]. Transmigration is mediated by the binding of αLβ2 to ICAM-1 and -2 [88]. The expression of VCAM-1 and ICAM-1 is upregulated in EAE [93]. CNS endothelial cells in EAE express CCL19 and CCL21, suggesting that these chemokines that are classically associated with LN trafficking are also involved in brain-homing in chronic inflammation [88]. CD8^+^ T cells in MS lesions show expression of αE-chain, suggestive of a tissue-resident memory phenotype, which increases in abundance with disease progression, and which allows these T cells to bind to E-cadherin-expressing T cells to form perivascular cuffs [94].

### Natalizumab

**Figure 2.**
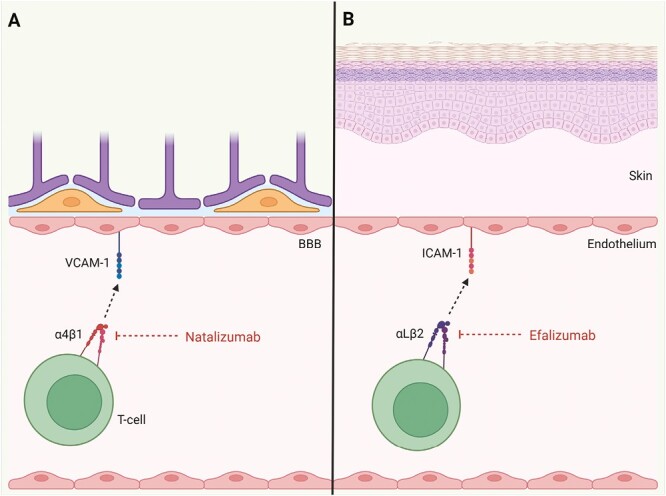
Targeting T-cell integrins in multiple sclerosis and psoriasis. **A**: Natalizumab inhibits the binding of α4β1 to VCAM-1 to inhibit extravasation of T cells across the blood-brain barrier (BBB). **B**: Efalizumab inhibits the binding of αLβ2 to ICAM-1 to inhibit the extravasation of T cells into skin. Created with BioRender.com

Initial studies of the effect of anti-integrin antibodies in EAE showed that only anti-α4β1 antibodies inhibited binding of lymphocytes to EAE vessels *in vitro*, prevented the accumulation of lymphocytes *in vivo*, and prevented the development of EAE [95]. By binding to α4β1 integrin and inhibiting its interaction with VCAM-1, natalizumab inhibits the initial binding of T cells to the endothelium of CNS blood vessels and thereby inhibits their infiltration of the CNS (Fig. 2A).

The AFFIRM trial, a phase-III RCT in patients with RRMS with at least one relapse in the prior year, showed that natalizumab was efficacious in reducing the annual relapse rate and the risk of sustained progression of disability, compared to placebo [35]. Natalizumab was furthermore shown to be efficacious in RRMS in combination with interferon β-1a [37]. These data support the paradigm of infiltrating lymphocytes being central to the pathophysiology of RRMS by showing that targeting this process affects disease severity [86]. In contrast, the ASCEND trial, a phase-III RCT in patients with SPMS for two years or more, showed that natalizumab was not efficacious in reducing disease progression at two years, measured by the Expanded Disability Status Score and Timed 25-Foot Walk; however, disease progression was slowed when measured using the 9-Hole Peg Test [36]. Thus, this failure of natalizumab in SPMS further reinforces the hypothesis that progressive MS is less reliant on lymphocyte infiltration and is instead driven by intra-CNS pathological processes behind a more intact BBB [96]. This is an instructive example of how the success or failure of a drug can inform the understanding of disease pathophysiology.

Following a series of case reports of PML [see Box 1] following treatment initiation, natalizumab was withdrawn from the market in 2005 but reintroduced in the USA and EU in 2006 [97]. Natalizumab is only available in the USA through a risk evaluation and mitigation strategy called the TOUCH Prescribing Program [67]. In the EU, it is authorized for use in highly active RRMS and rapidly evolving severe RRMS [98] but NICE recommends that it only be used in rapidly evolving severe RRMS [99]. Recommendations for reducing the risk of natalizumab-associated PML in MS patients include the routine use of anti-JCV antibody index testing and MRI screening [66]. Use of natalizumab may be justified in cases of co-morbid autoimmune and inflammatory diseases; one case report describes the sustained remission of multiple sclerosis, intestinal inflammation, and ankylosing spondylitis (AS) following the initiation of natalizumab after failed adalimumab therapy [100]. However, the efficacy of natalizumab in AS has not been established by RCTs.

## Psoriasis

Psoriasis is a chronic, autoimmune disease of the skin. The most common variant is psoriasis vulgaris, also known as plaque psoriasis, which affects 85–90% of patients and is characterized by raised, well-demarcated, erythematous plaques with adherent silvery scales, particularly on the elbows and knees [101]. A recent meta-analysis of the European cohort GWAS identified 63 psoriasis susceptibility loci, accounting for 28% of the estimated heritability; these loci include those associated with lymphocyte differentiation/regulation, the type-1 interferon pattern recognition pathway, regulation of the adaptive immune response, and regulation of the NK-κB cascade, and are most enriched among enhancers of Th1, Th17, and CD8^+^ T cells [102]. Interaction of these susceptibility loci with environmental factors, including skin trauma, infections, medications, and stress, may trigger psoriasis [103].

The immunological pathophysiology of psoriasis may be divided into initiation and maintenance phases. Injury to keratinocytes causes the release of nucleic acids and the anti-microbial peptide LL37, which form complexes to activate toll-like receptors in plasmacytoid and myeloid DCs, causing the release of IL-12 and IL-23 [104]. Following their migration to local LNs, activated DCs activate naïve T cells and promote the development of a skin-homing repertoire of adhesion molecules. Vitamin D metabolites induce the expression of CCR10 in T cells, which is the cognate receptor for CCL27, a chemokine that is selectively expressed by keratinocytes but does not enhance the expression of cutaneous lymphocyte-associated antigen (CLA) or CCR4, suggesting a more complex mechanism for the development of the skin-homing phenotype [105]. CLA is derived from post-translational modification of P-selectin glycoprotein ligand-1 and binds to E-selectin to mediate the rolling stage of T-cell extravasation in cutaneous venules [106]. CCR4 is present on nearly all skin-homing T cells and binds to CCL17 [106]. Extravasation of T cells is completed by the binding of α4β1 and αLβ2 to VCAM-1 and ICAM-1, respectively; αLβ2-ICAM-1 binding also mediates trafficking of T cells from the dermis to the epidermis [107]. IL-23 promotes the development of Th17 cells, which produce IL-17, shown to be a key cytokine in maintaining inflammation in psoriasis, and therapeutic inhibition of which has shown efficacy in resolving the pathological skin changes of psoriasis [108]. This has led to the current model of psoriasis as a Th17-mediated disease, rather than a Th1-mediated one, as was previously conjectured.

### Efalizumab

Efalizumab is a humanized anti-αL IgG1 monoclonal antibody. Efalizumab causes downregulation of αLβ2 expression by T cells and saturates the remaining surface αLβ2 molecules [109], thereby inhibiting the binding of αLβ2 to ICAM-1 to inhibit T-cell extravasation (Fig. 2B). Efalizumab caused reduced epidermal and dermal T-cell counts and increased circulating lymphocyte counts in a phase-II open-label trial in patients with moderate to severe psoriasis [109]. Consistent with the role of αLβ2 in the immunological synapse (reviewed by Walling and Kim [110]), efalizumab decouples the internalization of engaged TCR/CD3 complexes, possibly explaining the hyporesponsive state of T cells that occurs in efalizumab-treated psoriasis patients [111]. Efalizumab also decreases the infiltration of the lesional dermis and epidermis by CD11c^+^ DCs which express TNF-α and inducible nitric oxide synthase, two mediators of inflammation linked to disease activity [112]. In a phase-III RCT in patients with moderate to severe psoriasis, efalizumab was efficacious in reducing psoriasis area and severity, and maintaining this reduction, compared with placebo; no safety signals were identified [44]. However, the identification of three confirmed cases of PML [see Box 1] in patients treated with efalizumab for ≥3 years and no other immunosuppressants, two of whom died, led to the withdrawal of efalizumab from the market; it has not been subsequently reintroduced [45].

## Conclusion

The central role of T cells in IBD, MS, and psoriasis makes them enticing targets for targeted therapies for autoimmune and inflammatory diseases. Furthermore, the vital role of integrins in the multistep paradigm of extravasation that mediates the trafficking of T cells to sites of inflammation in these diseases, as well as their role in the action of T cells in the immunological synapse, mechanistically supports the targeting of T-cell integrins in autoimmune and inflammatory diseases.

In IBD, targeting the α4β7-MAdCAM-1 interaction with vedolizumab has shown efficacy in clinical trials and possesses an advantage with regard to safety due to the selectivity of said interaction to the gut, as does targeting β7-chain on its own with etrolizumab. Similar drugs, including ontamalimab, abrilumab, PN-943, and PTG-100, are in development and may meet an unmet need in patients for whom the current anti-integrin therapies are ineffective.

The efficacy of natalizumab in RRMS has not been replicated for SPMS and PPMS, possibly due to differences in the relative contribution of T-cell infiltration to CNS inflammation in each phenotype. Therefore, targeting T-cell integrins may naturally be a limited strategy in MS, necessitating the use of other drugs for the management of these more challenging phenotypes. Furthermore, the likely causative association of natalizumab with PML greatly reduces natalizumab’s utility by skewing the risk:benefit ratio out of its favour, such that its use in MS requires extensive risk evaluation and mitigation measures and its use in IBD is almost never appropriate.

In psoriasis, the originally promising drug efalizumab, which targets the αL-chain in αLβ2, has been withdrawn from the market due to its likely causative association with PML. Unlike α4β7, αLβ2 is not specific for the organ of interest, and therefore inhibition of αLβ2 was unlikely to be as successful.

In the future, more research should be carried out to investigate the reasons for the lack of response to therapies targeting T-cell integrins. Multiomics analysis to include genomic, transcriptomic, epigenomic, microbiomic, and metabolomic data may have a large part to play in this regard, as has been seen with the association of low serum vitamin D concentration with vedolizumab failure. Finally, Successful development and trials of orally and subcutaneously administered agents may negate the requirement for attendance to the healthcare setting for intravenous administration of currently available monoclonal antibodies, reducing the burden on patients.

## Data Availability

Not applicable.
